# Multimodal Imaging of Waldenstrom Macroglobulinemia-Associated Hyperviscosity-Related Retinopathy Treated with Plasmapheresis

**DOI:** 10.1155/2021/6816195

**Published:** 2021-12-15

**Authors:** Michael J. Schatz, Carl S. Wilkins, Oscar Otero-Marquez, Toco Y. P. Chui, Richard B. Rosen, Meenakashi Gupta

**Affiliations:** The New York Eye and Ear Infirmary, 310 East 14th Street, New York, NY 10003, USA

## Abstract

While plasmapheresis is well known to significantly improve both retinal findings and systemic manifestations associated with Waldenstrom macroglobulinemia, few reports exist documenting changes in optical coherence tomography angiography (OCT-A). The authors present a case of a patient with Waldenstrom macroglobulinemia who had resolution of white-centered peripheral retinal lesions and parafoveal outer nuclear layer hyperreflective material following plasmapheresis. Applying image analysis software to before and after OCT-A images, the authors were able to show an objective decrease in retinal capillary and large vessel density following plasmapheresis. This technique can be used to guide treatment and surveillance for patients with hyperviscosity-related retinopathy.

## 1. Introduction

Waldenstrom macroglobulinemia is a lymphoplasmacytic lymphoma, with an incidence of 2-5 cases per million person years [1], median age of diagnosis of approximately 69 years old, and a male-to-female ratio of approximately 2 : 1 [2]. It results in unregulated production of IgM which increases the viscosity of the blood. Hyperviscosity is thought to cause autoregulatory venous dilation, subsequent venous stasis, and a rise in intravascular pressure [3]. The resultant retinal hypoxia of the vascular endothelial cells leads to vascular tortuosity, retinal hemorrhage, exudates, and retinal vein occlusion. Previous studies have shown that as serum viscosity and serum IgM levels rise, both peripheral hemorrhages and venous dilation increases [3]. Though treating hyperviscosity improves the clinical appearance of the retina, some patients ultimately suffer from decreased vision due to ischemic maculopathy. OCT-A can aid in the noninvasive evaluation of retinal perfusion deficits and potentially guide treatment courses by monitoring microvascular integrity.

## 2. Case Report

**Figure 1:**
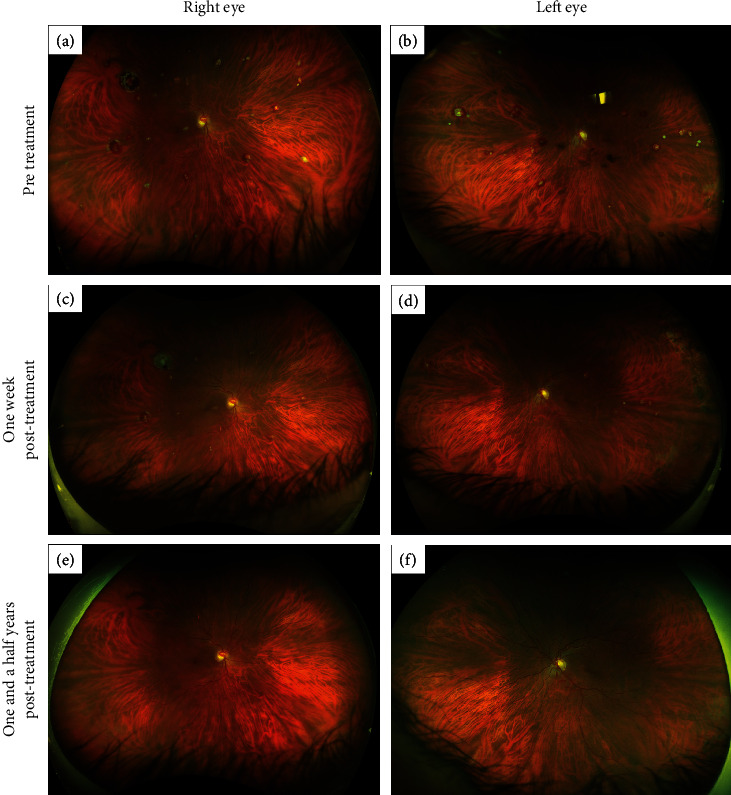
Fundus photos of right and left eyes. (a, b) Three days before beginning plasmapheresis. Prominent vascular tortuosity and white-centered peripheral lesions. Faint sclerotic vessels with box-carring-like changes apparent in the far nasal and temporal periphery of both eyes, possibly the result of pseudovasoocclusive events in the setting of venous stasis. (c, d) One week after finishing the third round of plasmapheresis. Dramatic improvement in the white-centered peripheral lesions is apparent. There is also a reduction in the box-carring-like changes in the periphery. (e, f) One and a half years later, there is sustained resolution of the white-centered peripheral lesions, but still some vascular tortuosity.

**Figure 2:**
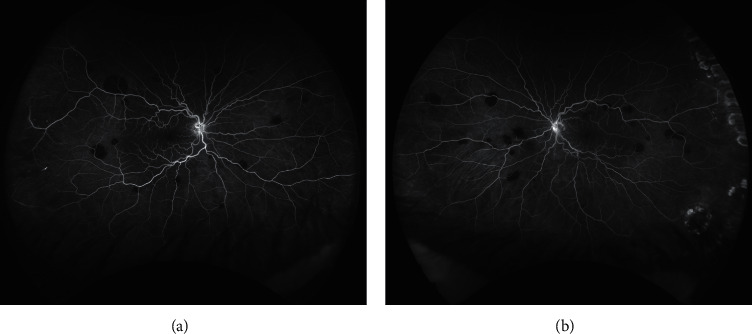
Fluorescein angiography of right (a) and left (b) eyes, three days before beginning plasmapheresis. Blockage is noted in areas of peripheral white-centered lesions. Capillary drop-out as well as peripheral staining are also apparent.

A 55-year-old man with an ocular history of posterior vitreous detachment, lattice degeneration with atrophic hole, and glaucoma suspect was referred for evaluation of retinal vascular tortuosity. Several months later, the patient was diagnosed by his oncologist with Waldenstrom macroglobulinemia. Upon reexamination, new vitreous hemorrhage, white-centered peripheral lesions, and increasing vascular tortuosity were noticed (Figures 1(a) and 1(b)). Fluorescein angiography from this time is shown in Figure 2. The patient also started to notice “waviness,” episodic dizziness, and headaches. His oncologist started one round of intravenous immunoglobulin, followed by three rounds of plasmapheresis.

**Figure 3:**
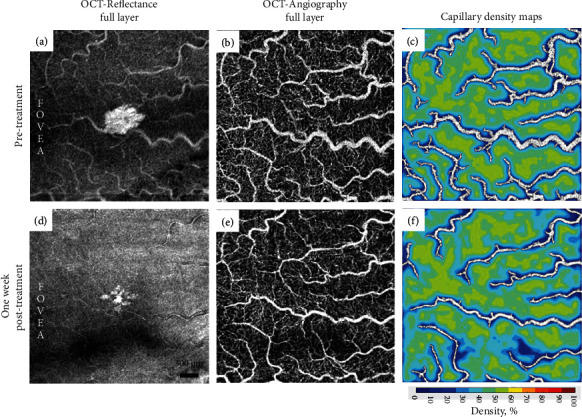
OCT angiography of the right retina, nasal to the fovea, before and one week after finishing plasmapheresis. (a, d) OCT reflectance full layer before (a) and after (d) plasmapheresis. One week after treatment, there was a significant decrease in the hyperreflective lesion nasal to the fovea. (b, e) Full vascular OCT-A layer before (b) and after (e) plasmapheresis. Qualitatively, there is a noticeable decrease in vessel diameter. (c, f) Capillary density maps before (c) and after (f) plasmapheresis. Capillaries are color-coded; large vessels are excluded and shown in white. After treatment, capillary density decreased from 47.62% to 45.35% and large vessel density decreased from 18.87% to 10.16%.

Pre- and one week posttreatment OCT-A scans nasal to the fovea were obtained with a commercial spectral domain OCT system (Avanti RTVue-XR; Optovue, Fremont, CA). The full retinal vascular layer OCT-A located between the inner limiting membrane and 9 *μ*m below the posterior boundary of the outer plexiform layer (Figures 3(b) and 3(e)) was used to measure the capillary and large vessel density. First, image registration was performed using ImageJ [4].

**Figure 4:**
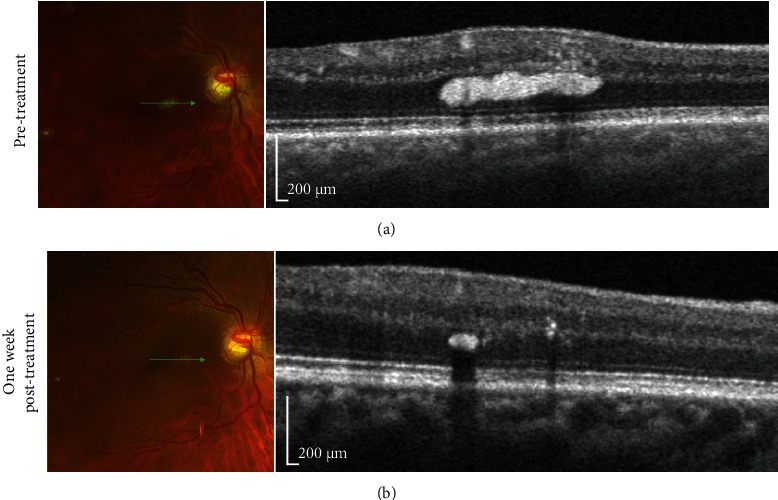
Color fundus photos and spectral domain OCT over white-centered peripheral lesions over the right nasal fovea three days before (a) and one week after (b) plasmapheresis show significant resolution of the outer nuclear layer hyperreflective material.

Segmentation of capillaries was performed after removal of the large blood vessels using local thresholding (MATLAB 2018b; MathWorks, Natick, MA) [5]. Next, computation of capillary and large vessel densities was performed. A color-coded density map was created for a rapid qualitative assessment (Figures 3(c) and 3(f)). After treatment, capillary density decreased from 47.62% to 45.35%, and large vessel density decreased from 18.87% to 10.16%. Pre- and posttreatment en face OCT (Figures 3(a) and 3(d)) and spectral domain OCT (Figures 4(a) and 4(b)) over the white-centered peripheral lesions showed significant resolution of the parafoveal outer nuclear layer hyperreflective material.

Following plasmapheresis, the patient noticed improvements in metamorphopsia, headaches, and dizzy spells. Visual acuity improved from 20/25 OU to 20/20 OU. The patient was started on five months of rituximab and bendamustine. The resolution of his white-centered peripheral lesions was sustained nearly one and a half years later (Figures 1(e) and 1(f)).

## 3. Discussion

We present a case of a patient with hyperviscosity-related retinopathy secondary to Waldenstrom macroglobulinemia that had dramatic improvement in both ocular and systemic symptoms following plasmapheresis. Plasmapheresis has been known for more than a decade to significantly reduce serum viscosity and hyperviscosity-related retinopathy. A 2008 case series of nine patients showed that plasmapheresis decreases the retinal venous diameter by an average of 15.3%, with those having the greatest reduction in serum viscosity showing the greatest reduction in retinal venous diameter [6]. One prior report of multimodal imaging in Waldenstrom macroglobulinemia-associated retinopathy demonstrated thickened choroid and shed retinal photoreceptors within serous retinal detachments and also showed a reduction in subretinal fluid and remaining outer retinal atrophy following local treatment with intravitreal bevacizumab [7].

Our patient did not have any associated serous detachment; however, serial imaging demonstrated a reduction in the average capillary diameter within the superficial plexus as well as a decrease in capillary and large vessel density following plasmapheresis. The decrease in vessel diameter is explained by the reduction in serum viscosity and hence a reduction in the venous stasis. A cause for the decrease in capillary and large vessel density, however, is less obvious. One possible explanation is that the hypoxia and pseudoocclusive events induced by the hyperviscosity led to the permanent destruction of capillary and large retinal vessels. Another explanation is that the reduction in capillary and large vessel density is artifactual, reflecting the decrease in vessel diameter more than a true decrease in the number of vessels. Even in the latter instance, the algorithm used in this report is still significant as it serves as an objective, proxy measurement for vessel diameter, which normally has only been compared qualitatively.

Long-term therapy traditionally consisted of R-CHOP (rituximab, cyclophosphamide, doxorubicin, vincristine, and prednisone) therapy, though recent studies show increased progression-free survival with bendamustine plus rituximab [8], which is what our patient received. The exact length of sustained improvement of hyperviscosity-related retinopathy following plasmapheresis, with or without adjuvant chemotherapy, as well as the frequency of intermittent therapy remains unknown. There is no standard of care established regarding how to monitor patients with hyperviscosity-related retinopathy undergoing treatment. OCT-A is a more sensitive modality to detect vascular integrity which may otherwise be missed with conventional imaging. We propose applying algorithms to OCT-A images to objectively measure the retinal vessel diameter and density to guide treatment duration and monitor for recurrence in patients with hyperviscosity-related retinopathy.

## Data Availability

Access to data is restricted to protect patient privacy but could be found in the New York Eye and Ear's electronic medical records.
